# Secreted cystatins decrease proliferation and enhance apoptosis of human leukemic cells

**DOI:** 10.1002/2211-5463.12958

**Published:** 2020-09-23

**Authors:** Samar Hunaiti, Hanna Wallin, Mia Eriksson, Marcus Järås, Magnus Abrahamson

**Affiliations:** ^1^ Division of Clinical Chemistry & Pharmacology Department of Laboratory Medicine Lund University Sweden; ^2^ Division of Clinical Genetics Department of Laboratory Medicine Lund University Sweden

**Keywords:** caspase‐3, cystatin C, cystatin D, cysteine peptidase, cysteine protease, protease inhibitor

## Abstract

Cysteine proteases are implicated in proteolysis events favoring cancer cell growth, spread, and death by apoptosis. Herein, we have studied whether the net growth and survival of the leukemic cell lines Jurkat, U937, and HL‐60 are affected by external addition of five proteins acting as natural cysteine protease inhibitors. None of the cystatins examined (A, C, D, and E/M) or chagasin showed consistent effects on Fas‐induced apoptosis when evaluated at 1 µm. In contrast, when the intrinsic apoptosis pathway was activated by hydrogen peroxide, addition of cystatin D augmented caspase‐3‐like activity within all three cell lines. Flow cytometric analysis of U937 cells also showed increased numbers of annexin V‐positive cells when hydrogen peroxide was used to initiate apoptosis and cells were cultured in the presence of cystatin D or C. Moreover, stimulation of hydrogen peroxide‐induced apoptotic U937 cells with either cystatin C or D resulted in a dose‐dependent decrease in the number of cells. Cell viability was also decreased when U937 cells were cultured in the presence of cystatin C or D (1–9 µm) only, demonstrating that these cystatins can reduce cell proliferation by themselves in addition to enhancing apoptosis induced by oxidative stress. These effects on U937 cells were paralleled by internalization of cystatins C and D, indicating these effects are caused by downregulation of intracellular proteolysis. External addition of cystatins C and D to HL‐60 and Jurkat cells demonstrated similar degrees of cystatin D uptake and decreased viability as for U937 cells, indicating that these effects are general for leukemic cells.

## Graphical abstract

The secreted cysteine protease inhibitors, cystatin C and cystatin D, were added externally to leukemic U937, Jurkat, and HL‐60 cell cultures. The cystatins were internalized into endo‐lysosomal vesicles resulting in augmented hydrogen peroxide‐induced apoptosis, as well as decreased proliferation, both in apoptotic and nonapoptotic cells.

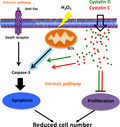

**Abbreviations**
ABTS: 2,2′‐azino‐bis(3‐ethylbenzothiazoline‐6‐sulfonic acid
APC: allophycocyanin
CLSM: confocal laser scanning microscopy
DAPI: 4′,6‐diamidine‐2′‐phenylindole‐dihydrochloride
FACS: fluorescence‐activated cell sorting
HRP: hoseradish peroxidase
MTT: 3‐(4,5‐dimethylthiazol‐2‐yl)‐2,5‐diphenyl‐tetrazolium bromide
NHMec: amino‐methyl‐coumarine
OPD: O‐phenylenediamine dihydrochloride
qRT–PCR: quantitative reverse transcription‐polymerase chain reaction
Z‐: carboxybenzoyl‐

Maintaining the balance between cysteine proteases and their inhibitors is crucial in the homeostasis of cellular processes such as antigen presentation, bone remodeling, wound healing, cell proliferation, motility, and apoptotic cell death [1, 2]. In disease, the ability of malignant cells to evade apoptosis is a hallmark of cancer [3]. Apoptosis is a physiological process with engagement of a number of cysteine proteases, including different caspases and cathepsins. An increased activity of papain‐like, lysosomal cysteine cathepsins of family C1 is often associated with cancer, and some evidence also refers to their involvement in apoptosis specifically. For instance, cathepsins B, H, K, L, and S may cleave the proapoptotic protein Bid to its active form, and simultaneously degrade the antiapoptotic molecules Bcl‐2, Bcl‐xL, and Mcl‐1. The release of cytochrome c from mitochondria is thereby triggered, and apoptosis initiation takes place [4, 5, 6]. The synthetic inhibitor of papain‐like proteases, E‐64d, has been shown to restrict Bid cleavage, thereby preventing apoptosis in various cancerous and noncancerous cell lines [6].

In humans, there are 11 well‐studied members of the cystatin protein family [7]. *In vitro* they are potent, reversible, competitive inhibitors of the different cysteine cathepsins in family C1, that is, cathepsins B, C, F, H, K, L, O, S, V, W, and X (or Z), normally present in the lysosomes of cells. Type 1 cystatins A and B (stefins A and B) are intracellular and found in the cytoplasm of most cells, and type 3 cystatins (L‐ and H‐kininogen) are intravascular inhibitors. The type 2 cystatins C, D, E/M, F, S, SN, and SA are secreted proteins and broadly found in body fluids, where they are supposed to constitute protection against enzymes leaking from damaged cells or those utilized by invading microorganisms [7, 8]. None of the cystatins inhibit caspases, the cysteine proteases in family C14, *in vitro* [4] but cystatins C, E/M, and F are also inhibitors of the lysosomal cysteine protease legumain or asparaginyl endopeptidase in family C13 [9].

We have in previous work shown that the two type 2 cystatins C and E/M are internalized by epithelial cancer cells of different origins and then colocalize with target enzymes in endo‐lysosomal vesicles. Intracellular activities of lysosomal cysteine cathepsins were downregulated following uptake, and both cellular migration and invasion in Matrigel were decreased [10, 11, 12]. The purpose of the present study was to examine effects of externally added type 2 cystatins on leukemic cells with respect to apoptosis, cell proliferation, and viability, with an overall aim to find new angles to suppress cell growth and viability in leukemia.

## Results

### Expression of type 2 cystatins and the Fas receptor in leukemic cell lines

Total RNA from Jurkat, HL‐60, and U937 cells was isolated and used for qRT–PCR to analyze the expression levels of the type 2 cystatins and the Fas receptor (CD95). The mRNA levels for cystatins C, D, E/M, F, S, SA, SN, and Fas were correlated with the expression of 18S rRNA. All cell lines expressed cystatins C and F (Table 1). The mRNA level was the highest for cystatin C, in all three cell lines. The cystatin F gene expression was highest in HL‐60 cells at a mRNA level 10‐fold higher than in Jurkat and U937 cells. Messenger RNA encoding cystatin D, E/M, S, SA, or SN could not be detected. This is in accordance with earlier work showing high‐level expression of cystatin C in different cell lines, as well as relatively high cystatin F expression in U937 cells [13]. A low‐level expression of mRNA encoding the Fas receptor was detected in all three cell lines (Table 1).

**Table 1:** Relative expression of type 2 cystatins and the Fas receptor (CD95) in Jurkat, HL‐60, and U937 leukemic cell lines. Total RNA was isolated, and the levels of mRNA encoding type 2 cystatins and Fas were measured by qRT–PCR in relation to 18S rRNA levels as endogenous control. Triplicate measurements of one cell culture experiment were performed. The mean ratio values shown are multiplied by a factor of 10^6^. Values denoted as zero were below the detection limit in the assay.

Protein (gene)	Cell line		
	Jurkat	HL‐60	U937
Cystatin C (*CST3*)	3742	3743	2534
Cystatin D (*CST5*)	1	0	0
Cystatin E/M (*CST6*)	1	0	0
Cystatin F (*CST7*)	47	628	63
Cystatin S (*CST4*)	0	0	0
Cystatin SA (*CST2*)	0	0	0
Cystatin SN (*CST1*)	0	0	0
Fas receptor (*FAS*)	15	6	10

### Screening for effects of externally added cystatins on apoptosis

To initiate the extrinsic or intrinsic pathways of apoptosis, we added either 0.2 µg·mL^−1^ of a monoclonal anti‐Fas antibody or 40 µm H_2_O_2_ to cultures of Jurkat, HL‐60, and U937 cells. Additionally, we added 1 µm of the intracellular inhibitor cystatin A or 1 µm of the secreted type 2 cystatins C, D, or E/M to the cell cultures. Chagasin, an endogenous inhibitor of the papain‐like cysteine protease in *Trypanosoma cruzi* (cruzipain), was also included in the experiment as an inhibitor of equal size as the cystatins but from a different protein family and hence structurally completely different [14]. Control cells were cultured in standard medium. Ongoing apoptosis was assessed by measurement of caspase‐3‐like activity in cell lysates by the fluorogenic substrate Z‐DEVD‐NHMec.

**Fig. 1:**
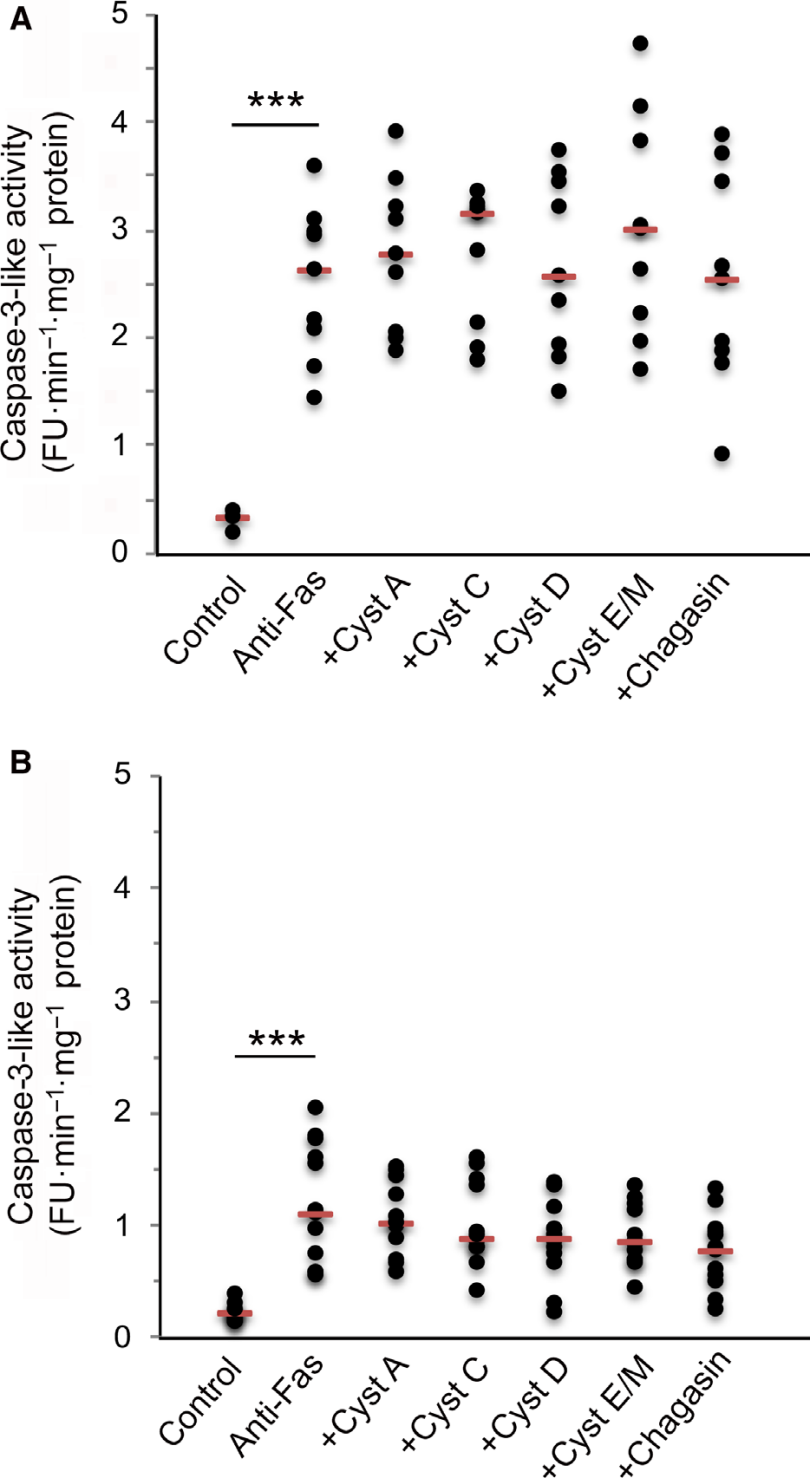
Effects of cystatins A, C, D, E/M, and chagasin on caspase‐3‐like activity in cells following activation of the extrinsic apoptosis pathway. An initial number of 500 000 Jurkat (A) or U937 (B) cells were seeded in 12‐well plates. The cells were incubated for 12 (Jurkat) or 15 (U937) h with 0.2 µg·mL^−1^ anti‐Fas as well as cystatins A, C, D, E/M, or chagasin at a final concentration of 1 µm. Control cells were incubated in standard medium. Caspase‐3‐like activity in cell homogenates was monitored by cleavage of the fluorescent substrate Z‐DEVD‐NHMec. Raw assay data from 3 to 4 independent cell experiments were grouped and are all shown, with red bars indicating median values for each group. Groups of data (8–16 data points, each being the mean result of three assay measurements) were compared and analyzed statistically by Mann–Whitney test. Significant differences between groups are indicated (***P < 0.001).

Incubation with anti‐Fas resulted in activated caspase‐3 in Jurkat and U937 cells after 12–15 h, but no significant caspase‐3 activity was observed in HL‐60 cells. Culturing in the presence of 1 µm of cystatins or chagasin showed no consistent effect on the caspase‐3‐like activity in any of the two leukemia cells further studied, when combined data from at least three independent experiments were analyzed statistically (Fig. 1).

**Fig. 2:**
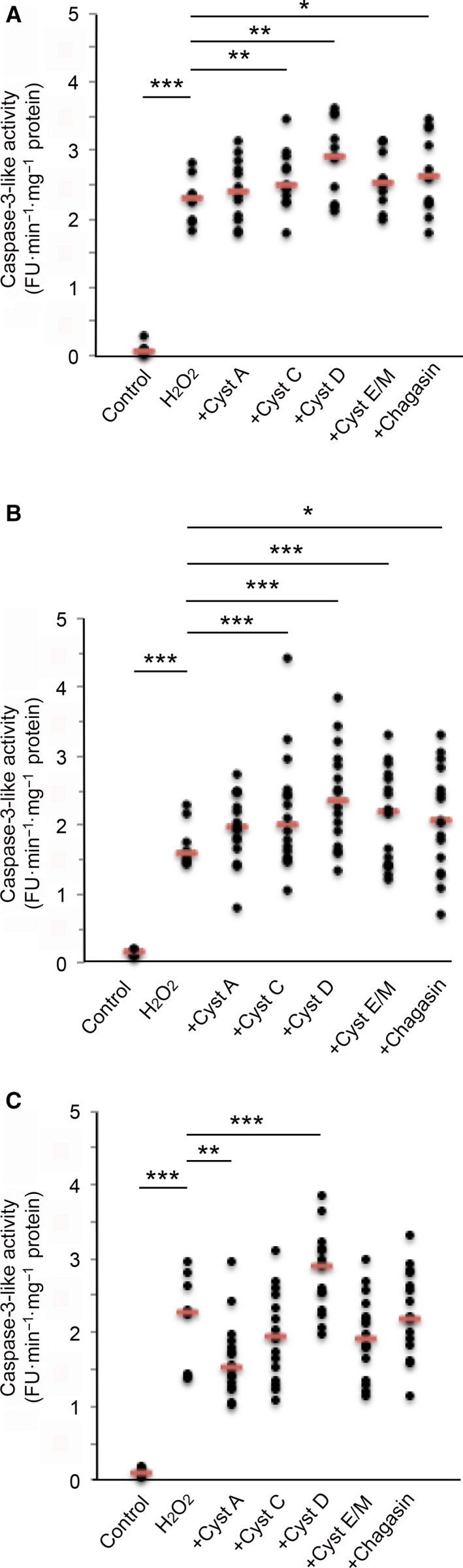
Effects of cystatins A, C, D, E/M, and chagasin on caspase‐3‐like activity in cells following activation of the intrinsic apoptosis pathway. A number of 500 000 Jurkat (A), U937 (B), or HL‐60 (C) cells were seeded into 12‐well plates. The cells were incubated for 12 (Jurkat) or 15 (U937 and HL‐60) h with 40 µm H_2_O_2_ and 1 µm cystatins A, C, D, E/M, or chagasin. Control cells were incubated in standard medium. Caspase‐3‐like activity in homogenates of the cells was monitored by cleavage of the fluorescent substrate Z‐DEVD‐NHMec. Raw assay data from 3 to 4 independent cell experiments were grouped and are all shown, with red bars indicating median values for each group. Groups of data (8–16 data points, each being the mean result of three assay measurements) were compared and analyzed statistically by Mann–Whitney test. Significant differences between groups are indicated (***P < 0.001; **P < 0.01; *P < 0.05).

When H_2_O_2_ was used to initiate the intrinsic pathway of apoptosis, caspase‐3‐like activity was increased in all three cell lines. The fluorescence signal was repeatedly seen augmented by simultaneous addition of cystatin D suggesting increased apoptosis, and despite a variation in levels of caspase‐3‐like activity between different independent experiments, the increase in activity could be seen also when raw activity data were analyzed without normalization to control (U937 and HL‐60, *P* < 0.001; Jurkat *P* < 0.01). Increased apoptosis was also seen when cystatin C or E/M was added to U937 cells (*P* < 0.001, both) and when cystatin C was added to Jurkat cells (*P* < 0.01). Cystatin A showed a reduction in caspase‐3‐like activity in HL‐60 cells (*P* < 0.01), but no trend toward apoptosis inhibition in Jurkat or U937 cells. The nonhuman inhibitor chagasin showed smaller additive effects on caspase‐3‐like activity in U937 (*P* < 0.01) and Jurkat (*P* < 0.05) cells (Fig. 2A–C).

**Fig. 3:**
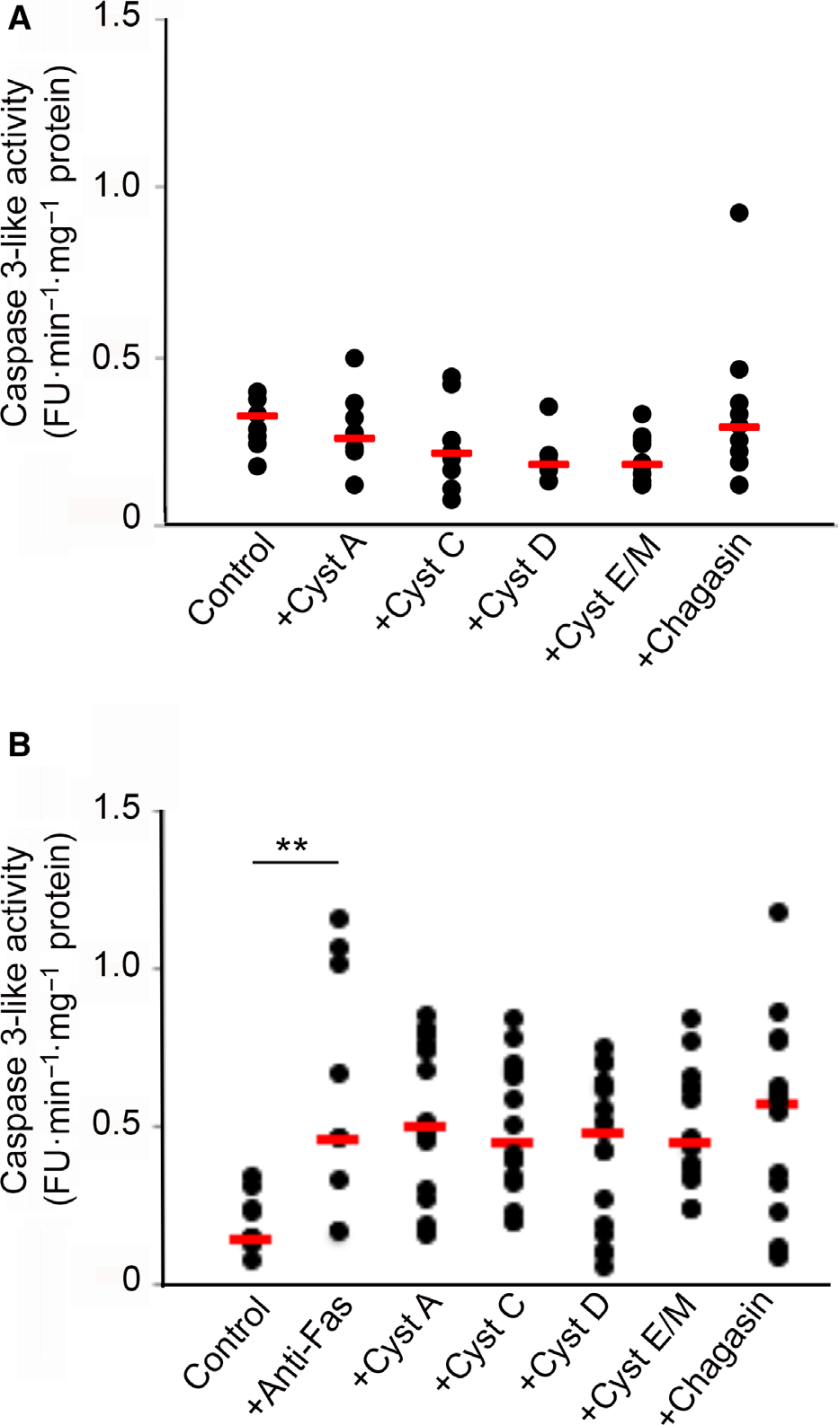
Effects of cystatins A, C, D, E/M, and chagasin to induce or inhibit caspase‐3‐like activity in (A) cells grown without apoptosis induction or (B) a homogenate of apoptosis‐induced cells *in vitro*. (A) U937 cells were incubated with medium containing 1 µm of cystatins A, C, D, E/M, or chagasin for 15 h, without simultaneous apoptosis induction. Control cells were incubated in standard medium. Caspase‐3‐like activity in homogenates of the cells was monitored by cleavage of the fluorescent substrate Z‐DEVD‐NHMec. (B) Cystatins A, C, D, E/M, or chagasin was added to a final 1 µm concentration, to a lysate of U937 cells in which apoptosis had been induced by incubation with an antibody against Fas for 15 h, prior to assay with the fluorescent substrate Z‐DEVD‐NHMec. Raw assay data from two independent cell experiments were grouped and are all shown, with red bars indicating median values for each group. Groups of data (4–8 data points, each being the mean result of three assay measurements) were compared and analyzed statistically by Mann–Whitney test (**P < 0.01).

As a control, we repeated the experiments without induction of apoptosis. Cystatins A, C, D, E/M, or chagasin were added at 1 µm final concentration to U937 cultures for 15 h, to find out if the inhibitors used could have any end effect on Z‐DEVD‐NHMec degradation by themselves. No increase in fluorescence was seen compared to control cells incubated in standard medium, suggesting that neither cystatin A, C, D, or E/M nor chagasin had any direct or indirect effects on proteases leading to Z‐DEVD‐NHMec hydrolysis in nonapoptotic cells (Fig. 3A). In another control experiment, we used a lysate of cells in which apoptosis had been induced for 15 h and added 1 µm of cystatins A, C, D, E/M, or chagasin to the caspase‐3 assay just before substrate addition. No significant effects were seen, indicating that living cells were a prerequisite for the boosted apoptosis and excluding that something in the cystatin preparations affected the assay results (Fig. 3B).

### Cystatins C and D increase apoptosis in U937 cells dose‐dependently

The unexpected results from the screening experiments using different cystatins, showing a small but significant augmentation of caspase‐3‐like activity, prompted us to further analyze cystatin effects on apoptosis, now measured as degree of annexin V staining. U937 cells were chosen, because the additional effects of cystatins on apoptosis were most pronounced in these cells. The inhibitors selected were cystatin D, as the effect was significant with this inhibitor in all cell lines, and cystatin C, also showing a consistent effect on apoptosis and, besides, being the most highly expressed inhibitor in the blood cells studied.

**Fig. 4:**
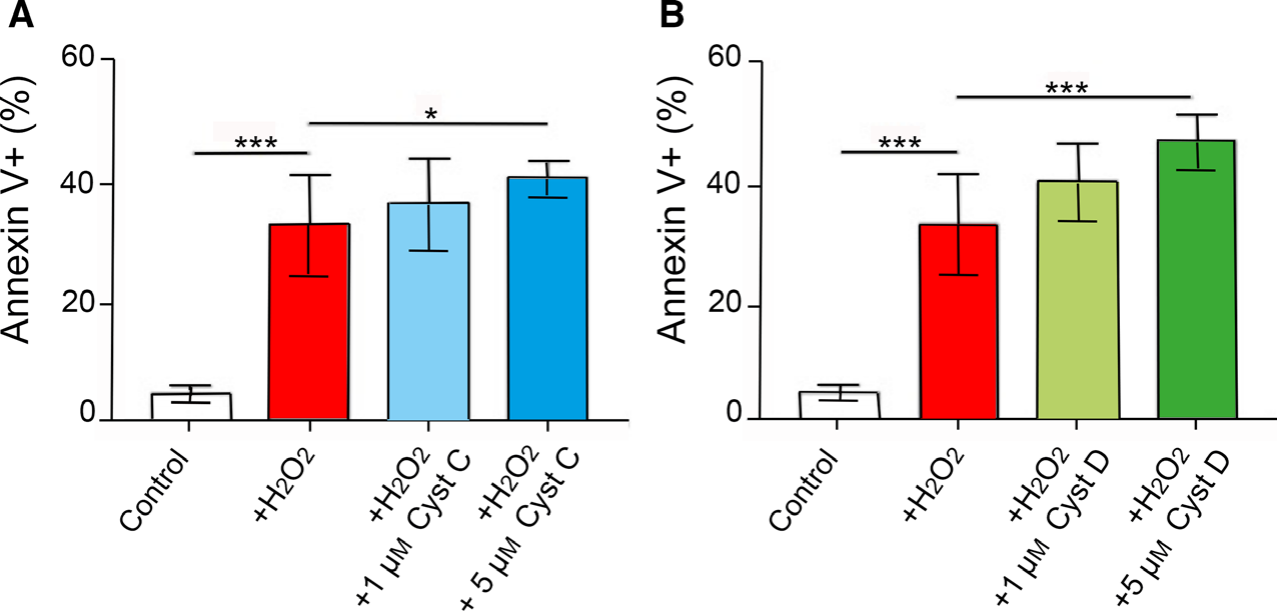
Augmented apoptosis by cystatins C and D seen by annexin V staining. U937 cells were incubated for 24 h with 40 µm H_2_O_2_ and 1 or 5 µm cystatin C (A) or cystatin D (B). Apoptosis was determined by flow cytometry using annexin V‐APC staining. Flow cytometry measurements were made in triplicate wells in three independent experiments. The bars show mean results, with error bars representing SD. Statistics were calculated by Mann–Whitney test (*P < 0.05; ***P < 0.001).

The U937 cells were incubated without or with addition of H_2_O_2_ to induce apoptosis and 1 or 5 µm cystatin C or D for 24 h. An increase in cell surface phosphatidyl serine signifying apoptosis was measured by fluorescently labeled annexin V and flow cytometry. Incubation with H_2_O_2_ under these conditions increased the amount of annexin V‐positive cells (*P* < 0.001) (Fig. 4) and simultaneous addition of 1 µm cystatin C or cystatin D to the cell culture medium further increased the amount of annexin V‐positive cells slightly. When the cystatin concentration was increased to 5 µm, the enhanced effects on apoptosis were more evident, especially when cystatin D was used (*P* < 0.001) (Fig. 4). Addition of the cystatins at the same concentrations without H_2_O_2_‐induced apoptosis induction of the U937 cells showed no significant effects on the amount of annexin V‐positive cells (data not shown).

### Cystatins C and D reduce viability of both apoptosis‐induced and noninduced U937 cells

The increase in apoptosis seen in Jurkat, HL‐60, and U937 cells when simultaneously incubated with H_2_O_2_ and cystatins C and D raised the question of the overall effects on cell quality. To investigate this, we used the MTT cell viability assay. Apoptosis was induced by H_2_O_2_ addition to U937 cells seeded into standard medium. Additionally, cystatin C or D (0, 1, 3, or 9 µm) was added to the medium before incubation of the cells for 48 h. The MTT result for the apoptosis‐induced control cells (H_2_O_2_ addition only) was set to 100% and the rest of the results were related to it.

**Fig. 5:**
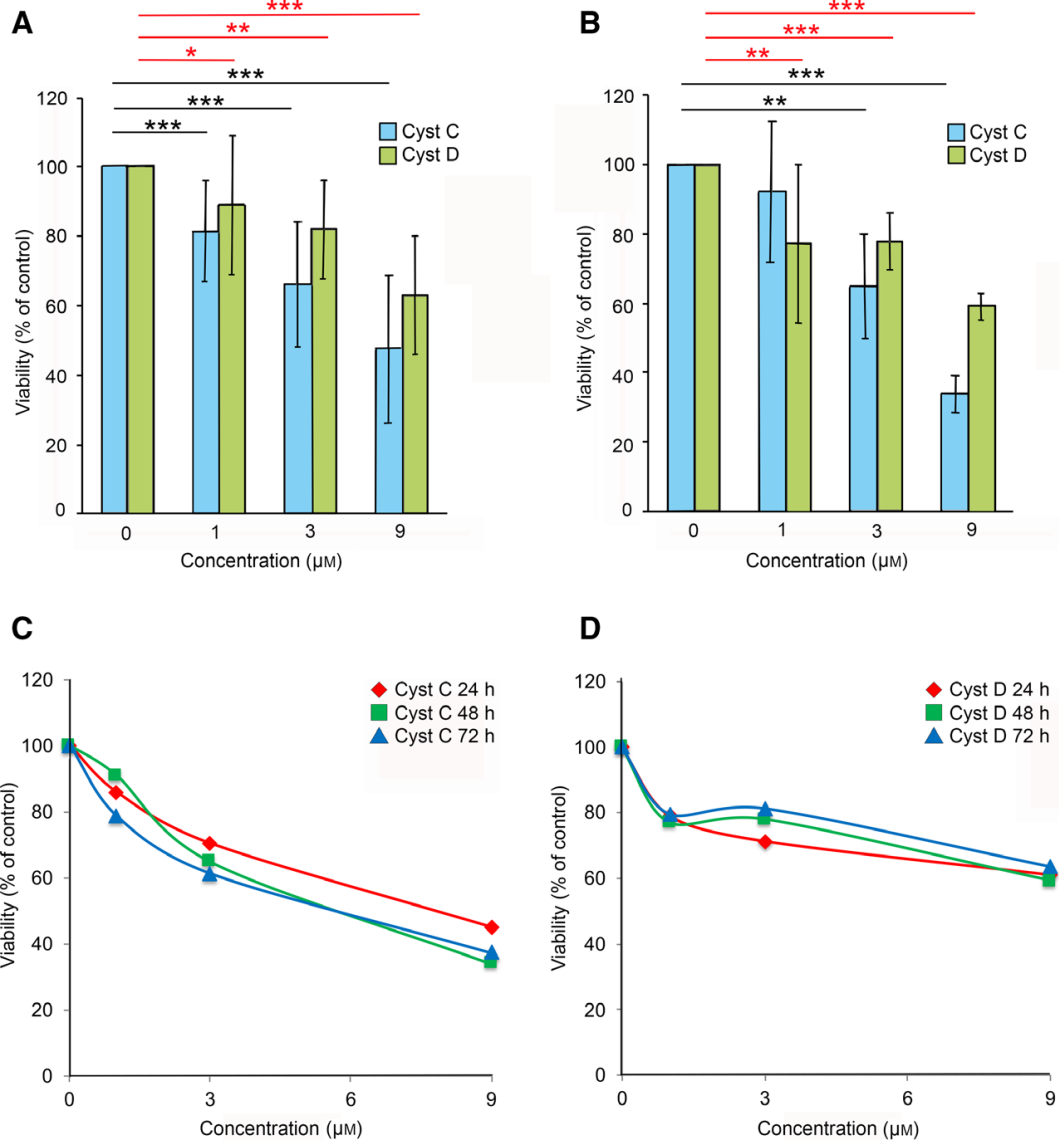
Cystatins C and D decrease U937 cell viability, both after apoptosis induction and in normal cells. U937 cells were incubated for 48 h with 0, 1, 3, or 9 µm cystatin C or cystatin D, and (A) with 40 µm H_2_O_2_ for apoptosis induction or (B) without apoptosis induction. For analysis of viability effects over time, U937 cells were incubated with 0–9 µm (C) cystatin C or (D) cystatin D without apoptosis induction for 24–72 h. After incubations with cystatins, MTT was added to the cell cultures for 4 h. Crystals formed in living cells were dissolved by DMSO, and the absorbance was measured at 540 nm. The results are expressed in relation to those for the control cells (0 µm cystatin). The mean ± SD for the mean results from three independent experiments are shown by bars in (A), (B). Statistics (9 data points/group) were calculated by Mann–Whitney test (***P < 0.001; **P < 0.01; *P < 0.05).) Errors bars for the SD of the mean values after 24, 48, or 72 h cultures shown in (C), (D) have been omitted as they overlap, indicating no significant difference in the relative viability of the cystatin treated cells at the different time points.

The viability of the apoptosis‐induced U937 cells decreased dose‐dependently as a result of both cystatins C and D addition. At 1 µm concentration of cystatin C, the number of living H_2_O_2_‐treated cells was reduced to 81% of the control cells (*P* < 0.01) and to 89% for cystatin D at the same concentration (*P* < 0.05) after 48 h. At 9 µm cystatin addition, the proportion of viable cells was reduced to 47% for cystatin C and to 63% for cystatin D (*P* < 0.001, both) (Fig. 5A).

We then performed a control experiment, adding the cystatins to U937 cells without apoptosis induction. The cell cultures were incubated in medium containing 0, 1, 3, or 9 µm cystatin C or D, but without H_2_O_2_. The viability of the control cells in this experiment was at a level approximately four times higher (*A*_540_, mean ± SD, 1.041 ± 0.127) than for the apoptosis‐induced control cells in Fig. 5A (*A*_540_, 0.248 ± 0.167). Interestingly, a similar relative decrease in viability as a result of cystatin addition was seen in these cultures of nonapoptotic cells as under apoptosis conditions. Addition of 1 µm cystatin C resulted in a slight reduction to 92% living cells, whereas the proportion of living cells was reduced to 76% after addition of 1 µm cystatin D (*P* < 0.01). When 9 µm cystatin C was added, the viability decreased to 34% of the control cells and the same concentration of cystatin D resulted in a viability reduction to 59% (*P* < 0.001) (Fig. 5B). The experiment was repeated at two additional time points, 24 and 72 h. The same dose‐dependent effect was seen independently of incubation time, when either cystatin C or D was used (Fig. 5C,D).

### Uptake of cystatins C and D in U937 cells

The results above indicate that cystatins have an antiproliferative effect on U937 cells and that apoptosis is augmented by cystatins C and D, when living cells are incubated with the inhibitors present in the surrounding medium. In previous work, we have seen internalization of the secreted type 2 cystatins C and E/M in epithelial cells [10, 11, 12, 15]. To address the uptake of type 2 cystatins also in blood cells, we analyzed the intracellular content of the cystatins after U937 cells had been cultured in cystatin C or D containing medium. The variants W106F‐ and (R24A,R25A)‐cystatin C were included in the experiment, as these variants have been used in another study where it was noted that W106F‐cystatin C resulted in an elevated uptake in MCF‐7 breast cancer cells compared to wild‐type cystatin C and much slower uptake was seen for (R24A,R25A)‐cystatin C [11]. The substitution of phenylalanine for the normal tryptophan at position 106 in the cystatin C molecule resulted in less efficient inhibition of cathepsin B and other papain‐like enzymes as this residue is located in a part of the molecule involved in binding of papain‐like enzymes [16], but legumain inhibition is not affected, as the legumain binding site is located in another part of the molecule [9]. The arginine residues at positions 24 and 25 in wild‐type cystatin C are not thought to participate in inhibition, of neither papain‐like enzymes nor legumain [11].

**Fig. 6:**
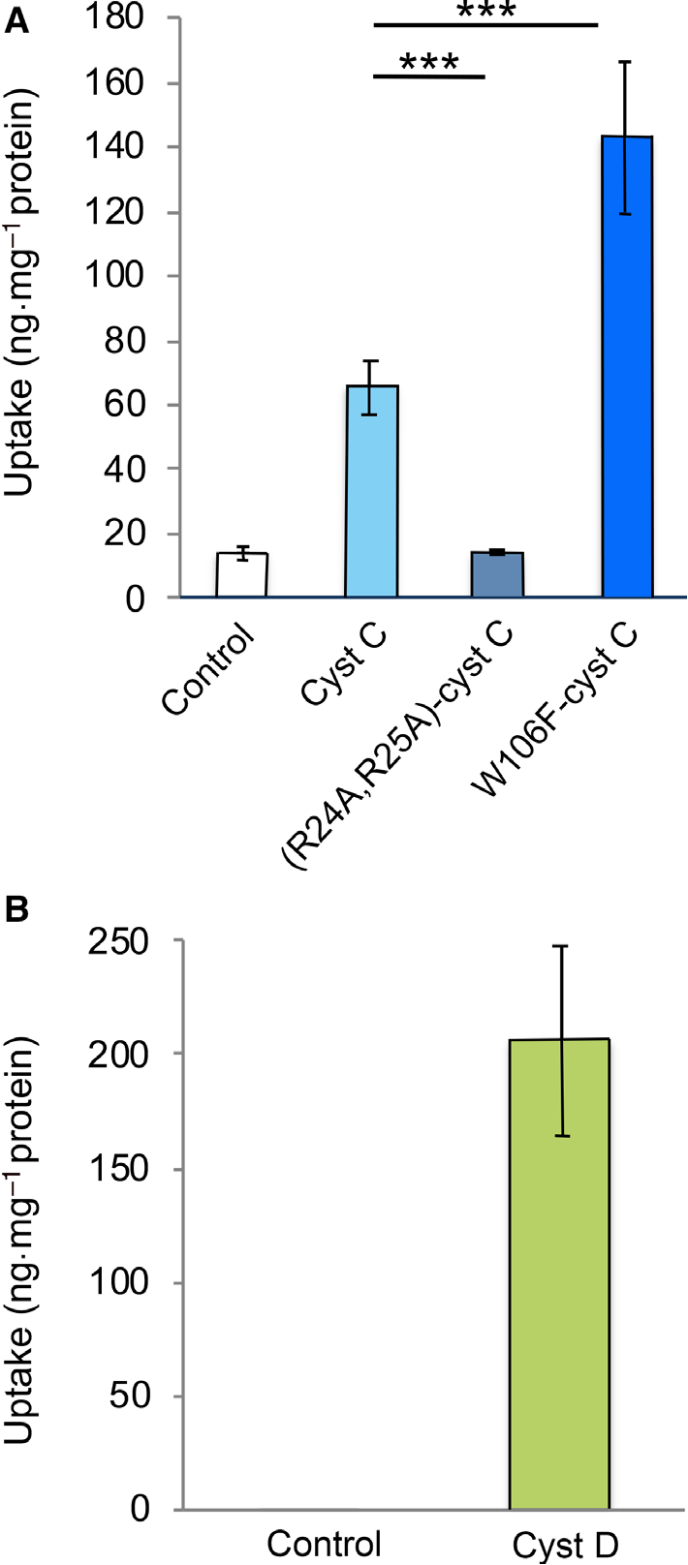
Uptake of cystatins in U937 cells. U937 cells were incubated for 5 h with 1 µm wild‐type cystatin C, (R24A,R25A)‐cystatin C, W106F‐cystatin C, or wild‐type cystatin D. Control cells were incubated in standard medium. The intracellular cystatin content was measured in lysates of the cells by ELISA assays specific for either cystatin C (A) or cystatin D (B). The measured values were correlated to the protein content of each lysate. Bars represent mean values of two independent experiments with four culture wells each. Error bars represent SD. Statistics were calculated by Mann–Whitney test (***P < 0.001). Cystatin D values for control cells were below the detection limit of the ELISA (< 0.26 ng·mg^−1^ protein).

U937 cells were incubated for 5 h in standard medium or medium with addition of 1 µm of the three cystatin C variants. Then, the cells were lysed and the content of cystatin C was measured by ELISA and related to the protein concentration in the lysate. The cystatin C content had increased 4.4‐fold from the endogenous 15 to 66 ng·mg^−1^ cell protein after incubation with wild‐type cystatin C compared to the control cells (*P* < 0.001). The increase in W106F‐cystatin C was 9.5‐fold (143 ng·mg^−1^ cell protein) compared to the control cells and doubled compared to wild‐type cystatin C (*P* < 0.001). The variant (R24A,R25A)‐cystatin C was not internalized during the 5‐h incubation (*P* = 0.86), in agreement with the uptake levels and internalization rates seen in MCF‐7 cells [11] (Fig. 6A).

Analysis of cystatin D uptake was performed by incubation of U937 cells with 1 µm cystatin D for 5 h before cells were lysed. To be able to measure the cystatin D content in the lysates, we used a new ELISA method (see Methods). The measured values of cystatin D were related to the protein content of the cell lysate. A high level of cystatin D, 206 ng·mg^−1^ protein, could be measured in lysates of cells incubated with cystatin D for 5 h, indicating a substantial uptake. The corresponding value for wild‐type cystatin C was 66 ng·mg^−1^ protein (Fig. 6A). The content of cystatin D in the control cells was below the ELISA detection limit (< 0.26 ng·mg^−1^ protein) (Fig. 6B).

### Intracellular localization of internalized cystatins C and D in U937 cells

**Fig. 7:**
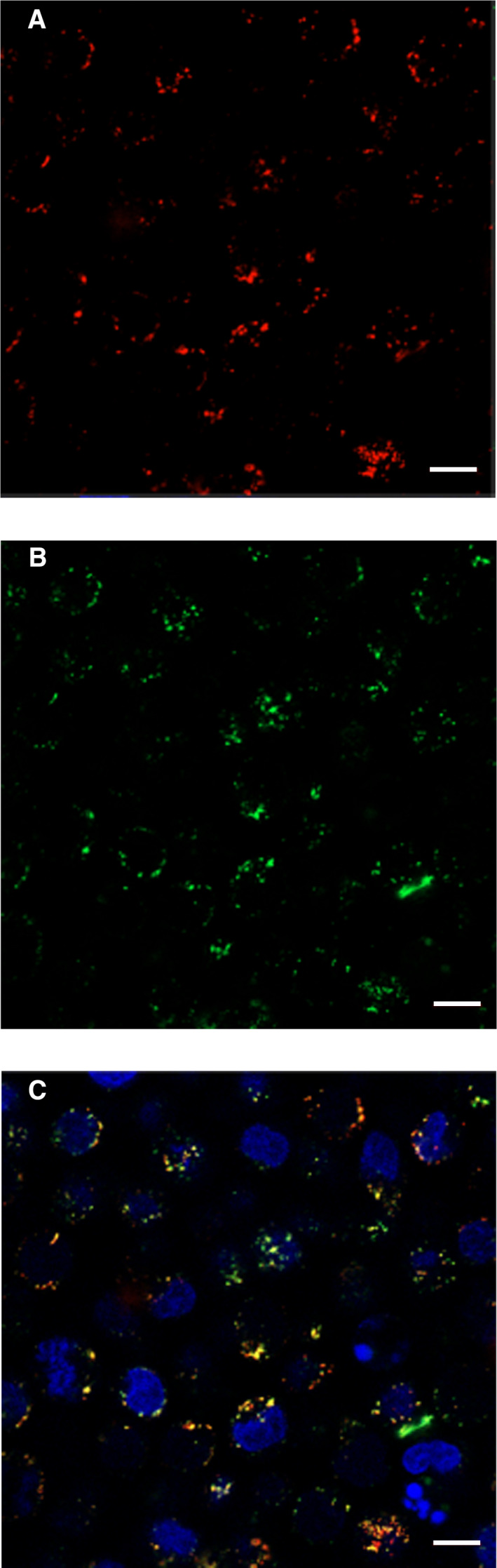
Intracellular localization of internalized cystatin C and cystatin D. U937 cells were incubated in medium containing both 3 µm Alexa Fluor 568‐cystatin C and Alexa Fluor 488‐cystatin D for 5 h. CLSM was used to visualize internalized cystatins in vesicular compartments. (A) cystatin C (red), (B) cystatin D (green) and (C) merge of (A) and (B) (yellow, colocalization). Nuclei were stained with DAPI (blue). Scale bar: 10 µm.

U937 cells were incubated with 3 µm cystatin C and/or cystatin D labeled with different fluorophores for 5 h before fixation and staining of nuclei with DAPI. Images were taken by CLSM using appropriate excitation and emission wavelengths for the fluorophores used. A significant uptake of both cystatins C and D was seen in all cells, in cytoplasmic vesicles resembling endo‐lysosomes. Colocalization of the two cystatins was seen when the images were merged and the red and green fluorescence resulted in yellow vesicles (Fig. 7A–C).

### Effect on U937 cell viability by variants of cystatin C

Stimulated by the verified uptake of wild‐type cystatins C and D in U937 cells seen by microscopy, we repeated the cell viability assay to address whether internalization is needed for the cystatin‐caused decreased viability we noticed earlier. Wild‐type cystatin C, (R24A,R25A)‐cystatin C, or W106F‐cystatin C was used for this purpose. Based on experience from the earlier experiments, we chose to incubate nonapoptotic U937 cells with medium containing each of the three cystatin variants at 3 µm concentration, and to incubate the cells for 48 h. The viability of the control cells incubated in standard medium was set to 100%, and the rest of the samples were compared to it.

**Fig. 8:**
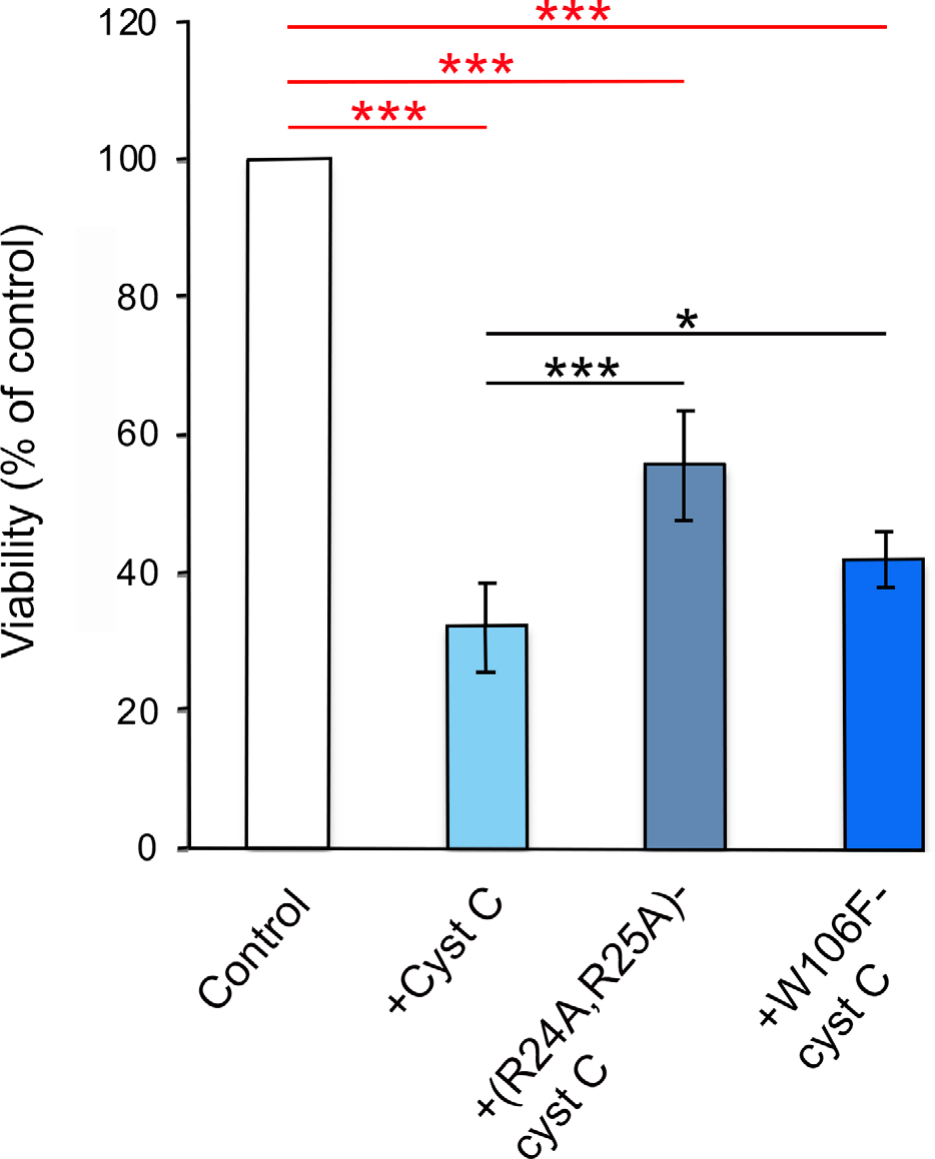
Effect of cystatin C variants on U937 cell viability. U937 cells were incubated with 3 µm wild‐type cystatin C, or any of the cystatin C variants (R24A,R25A)‐ and W106F‐cystatin C for 48 h. After incubations with cystatins, MTT was added to the cell cultures for 4 h. Crystals formed in living cells were then dissolved by DMSO, and the absorbance was measured at 540 nm. Measurements were made in two independent experiments, six wells each time, and results were related to the control cells. The bars show mean results, with error bars denoting SD. Statistics were calculated by Mann–Whitney test (*P < 0.05; ***P < 0.001).

The viability of U937 cells incubated with wild‐type cystatin C decreased to 34% (*P* < 0.001) in this experiment (Fig. 8), in good agreement with the earlier dose–response experiment (Fig. 5B). The effect of the cystatin C variants was less accentuated than that of wild‐type cystatin C but still showing reduction in viability, to 43% (*P* < 0.001) and 56% (*P* < 0.001) for W106F‐ and (R24A,R25A)‐cystatin C, respectively, compared to the control cells (Fig. 8). (R24A,R25A)‐cystatin C with a significantly decreased uptake rate in the U937 cells (Fig. 6A) showed a smaller effect on viability compared to wild‐type cystatin C (*P* < 0.001) (Fig. 8). The variant W106F‐cystatin C, with an increased uptake rate in the U937 cells (Fig. 6A) but with reduced capacity to inhibit papain‐like cysteine proteases, also showed less effect than wild‐type cystatin C in the MTT viability assay (*P* < 0.05) (Fig. 8).

### Viability effects of externally added cystatins C and D on HL‐60 and Jurkat cells

**Fig. 9:**
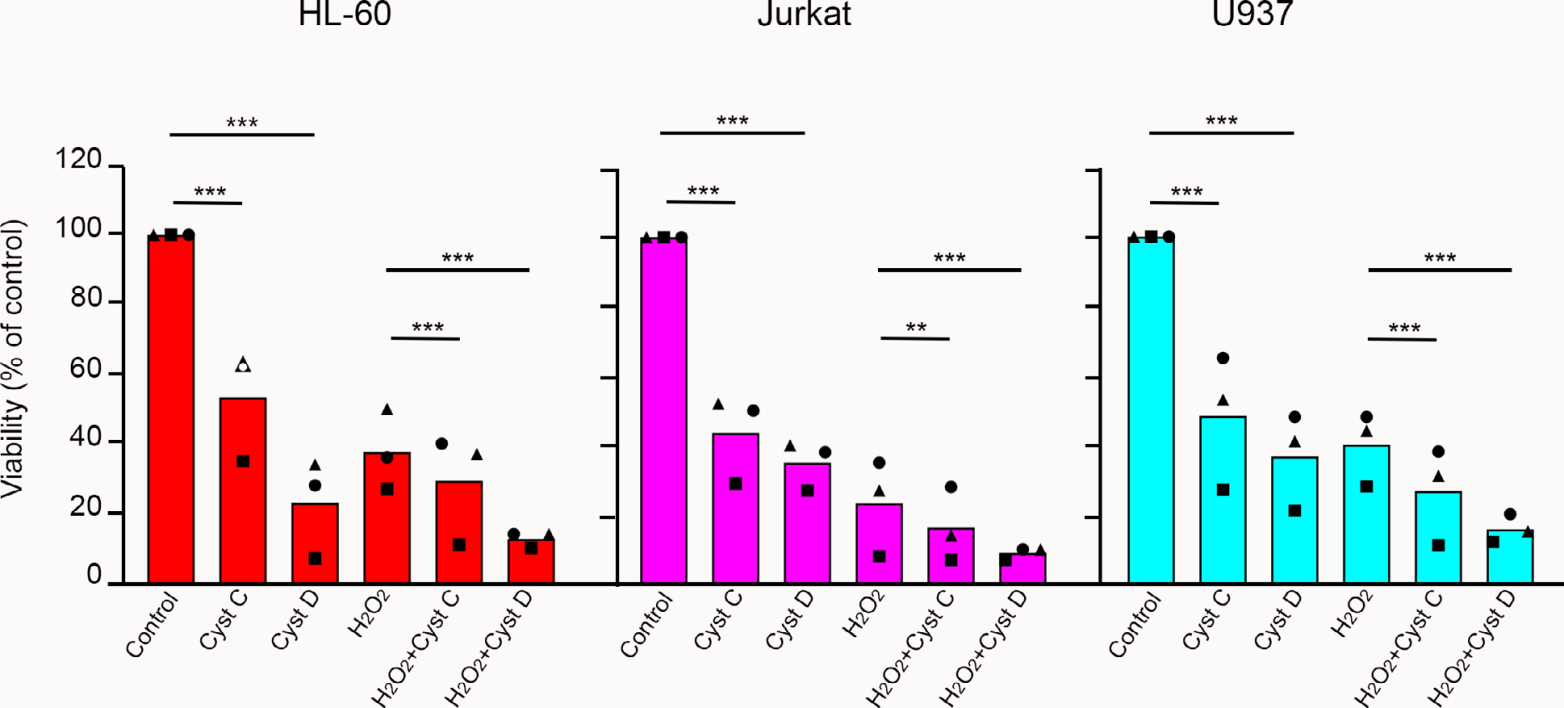
Effect of cystatin C and D on HL‐60, Jurkat, and U937 cell viability. HL‐60, Jurkat, and U937 cells were cultured under normal conditions or in medium containing 40 µm H_2_O_2_ to induce apoptosis. Cystatin C or D (3 µm) was externally added to the cell cultures 48 h before MTT assay. Experiments were made on three independent occasions, and 16–24 individual wells were analyzed for each condition, each time. The mean result of the control cells in each experiment was set to 100%, and the rest of the values were correlated with that. Bars illustrate mean values of all three experiments, and symbols represent mean results for each experiment (triangles, experiment 1; squares, experiment 2; circles, experiment 3). Statistics were calculated by Mann–Whitney test on groups of raw data (**P < 0.01; ***P < 0.001).

The results for U937 cells collectively demonstrate a marked effect of both cystatins C and D on proliferation, which is paralleled by a cellular uptake of the protein inhibitors. To elucidate if the viability decrease is general in leukemic cells, we conducted a parallel study of the three cell lines used in our initial screen of apoptosis effects by externally added cystatins. Cystatin C or D was added to the culture medium of HL‐60, Jurkat, and U937 cells to 3 µm final concentration before incubation for 48 h, both in the absence and in the presence of 40 µm H_2_O_2_ to induce apoptosis. The combined results from three experiments showed that the viability of HL‐60, Jurkat, and U937 cells was affected in a similar way by cystatin C, with a mean reduction in viability to 51%, 42%, and 44% of control, respectively (*P* < 0.001 for all; Fig. 9). The effect of externally added cystatin D was more pronounced on all three cell lines, with mean numbers of viable cells being 21% of control for the HL‐60 cells, and 34% for the other two cell lines (*P* < 0.001 for all). Induction of apoptosis caused a mean reduction in viability after 48 h to 37% and 38% of control for HL‐60 and U937 cells, respectively, and to 20% for Jurkat cells. External addition of 3 µm cystatin C caused a further reduction of HL‐60 and Jurkat cell viability to 26% and 14% of control, compared to 24% for U937 cells (*P* < 0.001 for HL‐60 and U937, *P* < 0.01 for Jurkat; Fig. 9). The mean effect of 3 µm cystatin D addition when cells were stressed by H_2_O_2_ was again more pronounced. The cell viability decreased to 12% of control for HL‐60 cells, and 15% and 8% for U937 and Jurkat cells, respectively (*P* < 0.001 for all). Taken together, a trend toward a larger effect on cell viability by 3 µm cystatin D than by the same concentration of cystatin C was seen both under cellular stress and in normal culture for HL‐60 and Jurkat, in agreement with the observations from dose–response experiments on U937 cells (Fig. 5). The overall conclusion from the comparison of the HL‐60, Jurkat, and U937 cells was that the cells were similarly affected by 3 µm cystatin C or cystatin D, both under apoptotic and normal conditions.

**Fig. 10:**
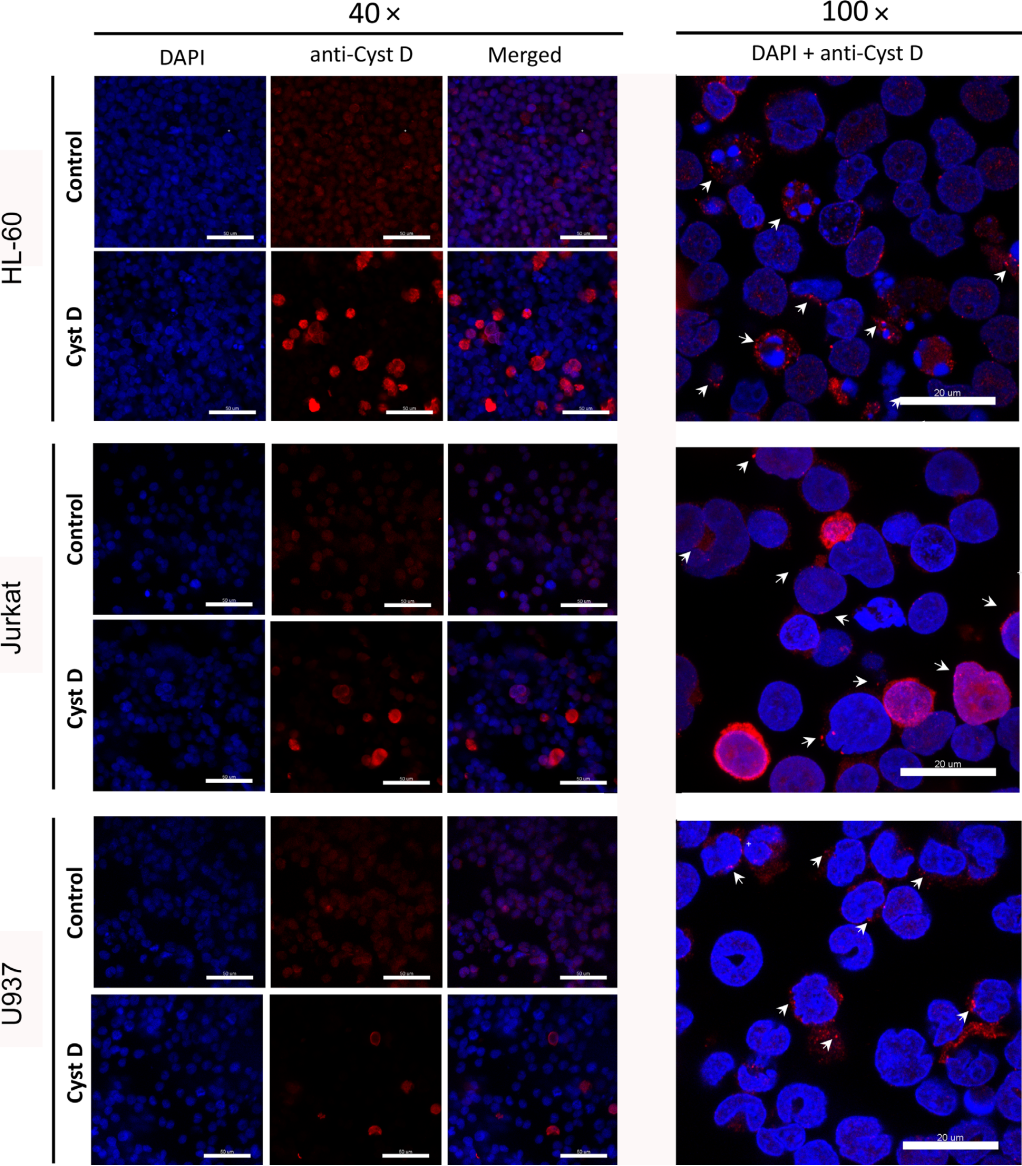
Internalization of cystatin D in leukemic cells. HL‐60, Jurkat, and U937 cells were incubated with 5 µm cystatin D for 5 h before fixation and immunostaining with a polyclonal antiserum directed against human cystatin D. CLSM was used to visualize the internalized cystatin D (red). Nuclei were stained with DAPI (blue). White arrows highlight staining in vesicular compartments. Scale bars: 50 µm (40× images) or 20 µm (100× images).

For U937 cells, we addressed the uptake of cystatin D from the culture medium by studying the cellular localization of fluorescently labeled cystatins C and D (Fig. 7) and concluded that the two inhibitors seem to follow the same internalization route ending in vesicles in the endo‐lysosomal system. Externally added cystatin C is known to get internalized into several cell types [10] but less is known about cystatin D. As the three leukemic cells under study do not express cystatin D, we could use immunostaining of the cells to compare the uptake. Parallel cultures of HL‐60, Jurkat, and U937 cells were incubated with 5 µm cystatin D for 5 h. In all three cell lines, a polyclonal antiserum raised against recombinant cystatin D revealed specific staining in a granular pattern in the cytoplasm, agreeing with an import of the cystatin into endo‐lysosomal vesicles (Fig. 10).

## Discussion

Cystatin C is produced by most cells in mammals and as it is a secreted protein it can be found in all body fluids (at concentrations up to several μm), where it is presumed to play its biological role as an efficient cysteine protease inhibitor [8]. The expression of other secreted type 2 cystatins is more restricted. Cystatin D, for example, is mainly expressed in parotid and tear glands, and consequently, it is found in saliva and tears in relevant concentrations [17]. We have earlier observed a cellular uptake when the type 2 cystatins C and E/M were added in μm concentrations to cultures of different epithelial cancer cells, with parallel effects on cellular properties such as migration [10, 11, 12]. At the organism level, such uptake into epithelial cells following intraperitoneal or intravitreal injection of physiological amounts of human cystatin C was early reported [18]. Other examples of presumably intracellular effects reported as consequences of externally added type 2 cystatins include those for chicken cystatin to inhibit poliovirus replication [19], human cystatin C to inhibit herpes simplex virus replication [20] and virus‐induced apoptosis [21], and human cystatins C and D to inhibit coronavirus replication [22, 23]. These effects were seen in various cells of epithelial origin, but there is also evidence for uptake and subsequent intracellular functions of type 2 cystatins in cells of the immune system (reviewed in Ref. [24]) more closely related to the promyelocytic (HL‐60 and U937) or lymphocytic (Jurkat) cells used in the present study. Cystatin C is, for example, taken up by human macrophages [25], and externally added cystatin C has been reported to affect the viability of tuberculosis bacteria in infected macrophages [26] as well as the control of antigen presentation by dendritic cells [27, 28, 29]. Thus, there is ample evidence indicating that secreted cystatins could also have biological functions intracellularly, in many different cell types.

A molecular mechanism leading to uptake of the type 2 cystatins to allow intracellular functions within various cell types has to our knowledge not been characterized yet, but in the case of rat kidney it has been demonstrated that the megalin receptor is involved and responsible for the resorption of cystatin C and other proteins in proximal tubule cells [30, 31]. The leukemic cells studied here do not express the megalin receptor, however, so additional internalization routes must be at hand. One type 2 cystatin not addressed in our study is cystatin F [32], which is specifically expressed in blood cell lineages and found intracellularly in U937 cells [13, 33]. Cystatin F is a glycoprotein and is specifically directed to vesicular compartments by mannose‐6‐phosphate tagging (reviewed in Ref. [34]). The inhibitor can thus be retained within cells following its production, but could also specifically be taken up by mannose‐6‐phosphate receptors on the surface of immune cells [35, 36]. Cystatins C and D are not glycoproteins, and the cystatins used in the present study were all recombinantly produced in *E. coli*, so their uptake need to be explained by another mechanism, however.

The lysosomal cysteine cathepsins that are efficiently inhibited by the secreted cystatins *in vitro* are involved in diverse cellular processes including apoptosis. Using neuroblastoma and breast cancer cell line models, Droga‐Mazovec *et al*. [6] demonstrated that the synthetic, membrane‐permeable cysteine cathepsin inhibitor, E64d, inhibited apoptosis. Our initial hypothesis in the present study was that apoptosis inhibition could also be a consequence of cellular cystatin uptake. We therefore performed a screening experiment with five highly purified, recombinantly produced protein inhibitors of cysteine cathepsins, including cystatins C and E/M for which we had earlier internalization results. The cells chosen for study were three leukemic cell lines, as commonly used model cells for apoptosis‐related mechanistic studies [37]. Contrary to our expectations, some of the cystatins showed a consistent effect to augment caspase‐3‐like activity when the leukemic cell lines were cultured in medium containing μm concentrations of cystatins (Fig. 2). This effect may be cell type specific, but could also reflect the more specific route of cystatin internalization into a distinct cellular compartment, compared to a membrane‐permeable nonprotein inhibitor which likely distributes widely in the cell. According to our microscopy experiments (Figs 7 and 10), this cellular compartment seems to be one in the endo‐lysosomal system.

Following the initial screening experiments to elucidate possible apoptosis effects, we focused our studies on cystatins C and D. These inhibitors evidently affect the overall rate of leukemia cell growth dose‐dependently under conditions of oxidative stress leading to apoptosis (Fig. 5A), but also when cells were not subject to oxidative stress induction (Figs 5B and 9). Previous studies of effects on the cellular level by externally added type 2 cystatins have indicated growth promotion of fibroblasts by the chicken analogue to human cystatin C [38], but growth inhibition of melanoma cells by a recombinant snake venom cystatin [39]. These opposing results could reflect different inhibition profiles of the cystatins used or, alternatively, that various cell types are differently affected by cystatins. The latter option should be important to follow up on, as would the question of relative cystatin effects on normal and cancerous cells. This needs to be done before we can assess the potential of our results for human cystatins on human leukemia cells, for an approach to decrease cancer growth by directed cellular cysteine protease inhibition.

The combined data from caspase‐3 and annexin V assays (Figs 2 and 4) indicate that cystatin D has especially large effect on apoptosis in the leukemic cells studied. Interestingly, this inhibitor is not expressed at all in the leukemia cells, but when internalized it reached even higher concentrations than the endogenous levels of cystatin C or the concentrations found after uptake (Fig. 5). Different profiles of target enzyme inhibition for cystatins C and D [40] could explain the effect on apoptosis, but the relatively larger increase in intracellular concentration of cystatin D could provide another explanation. The latter explanation gains some support from a similar observation in melanoma cells, showing a low degree of uptake of cystatin E/M but large effects on cellular migration compared to cystatin C [12]. Like cystatin D in the leukemia cells studied here, cystatin E/M was not expressed in the melanoma cells. Yet, the net effect on the cells following internalization was larger than seen for cystatin C, which was both internalized and endogenously expressed in the melanoma cells [12]. Internalization into different cellular compartments seems to be a less likely explanation for the results in the present study, since cystatins C and D appear to colocalize intracellularly following uptake (Fig. 7).

In an attempt to shed light on the mechanism by which cystatins C and D reduce cell proliferation, we studied the relative effects on cell viability by two variants of cystatin C with altered rate of cellular uptake compared to wild‐type cystatin C (Fig. 8). The results support that internalization is a prerequisite for the leukemia cell growth inhibition since the variant (R24A,R25A)‐cystatin C, which is taken up less efficiently (Fig. 6) but has the same enzyme inhibiting properties as wild‐type cystatin C [11] shows significantly smaller effects on cell viability. The variant W106F‐cystatin C, on the other hand, did not show enhanced growth inhibitory effects despite showing an increased rate of internalization (Fig. 6) as expected. Rather, the effect was lower than that seen for wild‐type cystatin C (Fig. 8), which could be explained by the generally decreased affinity for cysteine cathepsins W106F‐cystatin C shows [11]. This in turn highlights the important next question to address, that of which enzyme of the dozen candidates in the C1 and C13 families of cysteine proteases is primarily affected by type 2 cystatin uptake. Future work to pinpoint the target enzyme to unravel the mechanism for leukemia cell growth inhibition should be pursued.

Our data supporting a suppressive effect by cystatin D on leukemia cell proliferation agree well with a study on colon carcinoma cells [41], demonstrating that cystatin D has tumor suppressor activity in this cell type. In a series of elegant experiments, it was demonstrated that cystatin D had effects on several cellular events, including proliferation, migration, and expression of cancer‐promoting genes. The authors concluded that these effects were due to upregulated intracellular cystatin D but also that some of the effects seen, including decreased proliferation, could be independent of its protease inhibitory activity. Additionally, some evidence was presented to indicate that externally added cystatin D did not cause suppression of proliferation [41]. The latter contrasts to our results in leukemia cells and stresses that more work is needed, both to address which target enzyme(s) is affected by cellular uptake of type 2 cystatins and how cell type‐specific their effects on cancer cell growth are.

## Methods

### Production, purification, and characterization of proteins

Recombinant cystatins C and D were expressed in *Escherichia coli*, purified, and characterized according to protocols described in detail elsewhere [17, 42]. The W106F‐ and (R24A,R25A)‐cystatin C variants were produced by site‐directed mutagenesis with plasmid pHD313 as a template for thermal cycling with primers encoding the mutations and expressed, purified, and characterized in a similar way as the wild‐type cystatins [11, 16]. Cystatin A was expressed in *E. coli* using plasmid pHD389 as described for wild‐type cystatin D [17] and chagasin using a pGEX expression plasmid as described in detail earlier [14].

Butyl‐S Sepharose™, Q‐Sepharose™, or Sephadex™ (GE Healthcare LifeSciences AB, Uppsala, Sweden) was used to purify the expressed proteins depending on the properties of the protein [11] before quality was controlled by agarose gel electrophoresis at pH 8.6 and SDS/PAGE (NuPAGE, 4–12% Bis‐Tris; Invitrogen Life Science, Grand Island, NY, USA). Finally, as a last purification step the recombinant proteins were applied to Detoxi‐Gel™ Endotoxin Removing Gel (Pierce, Rockford, IL, USA). Protein concentrations were determined by Coomassie Protein Assay (Thermo Fisher Scientific Inc., Rockford, IL, USA) according to the manufacturer's recommendations and by measurement of *A*_280_ (NanoDrop 2000; Thermo Fisher Scientific Inc.). All recombinant proteins used were at least 90% pure and fully active as protease inhibitors according to papain titration [43].

### Cell culture

The cell lines Jurkat (human leukemic T cells), HL‐60 (human promyelocytic leukemia), and U937 (human histiocytic lymphoma) were cultured in RPMI 1640 medium (Life Technologies Ltd, Paisley, UK) supplemented with 10% (v/v) fetal bovine serum (Life Technologies) and antibiotics (10 U·mL^−1^ penicillin and 10 µg·mL^−1^ streptomycin; Life Technologies) at 37 °C in an incubator with 5% CO_2_, in culture flasks (Nunc A/S, Roskilde, Denmark). All cell lines were purchased from ATCC (LGC Standards GmbH, Wesel, Germany). The cells were regularly passaged three times a week using standard *in vitro* cell culture techniques.

To prepare cell lysates, cells were first pelleted and washed with PBS without calcium or magnesium (Life Technologies). After the washing step, cells were lysed with 0.2% (v/v) Triton X‐100 (Sigma‐Aldrich, Steinheim, Germany) in PBS. Eventually, a protease inhibitor cocktail was added to cell lysates to result in final concentrations of 5 mm benzamidinium hydrochloride, 15 mm NaN_3_, and 10 mm EDTA.

### Quantitative reverse transcription‐polymerase chain reaction

The expression of mRNA for the type 2 cystatins C, D, E/M, F, S, SA, and SN as well as the Fas receptor (CD95) was measured in the three blood cell lines. TRIzol reagent was used for isolation of total RNA according to the manufacturer's recommendations (Invitrogen). Additional purification of the RNA was performed by the RNeasy Mini kit (Qiagen AB, Solna, Sweden). Then, cDNA was synthesized from 25 ng of total RNA according to the manufacturer's guidelines. The quality and the concentration of the isolated RNA were determined by electrophoresis in a nondenaturing agarose gel and by measurement of the *A*_260_/*A*_280_ ratio (NanoDrop 2000; Thermo Fisher Scientific) as earlier described [12]. A StepOnePlus real‐time PCR device (Applied Biosystems, Foster City, CA, USA) was used for the thermal cycling, and all reagents for quantitative reverse transcription‐polymerase chain reaction (qRT–PCR) were from Life Technologies.

### Fluorometric measurement of caspase‐3‐like activity

Following initial optimization of incubation time and concentration of apoptosis inducers, Jurkat, HL‐60, or U937 cells were seeded at a density of 500 000 cells·mL^−1^ in 12‐well cell culture plates and incubated for 12 h (Jurkat cells) or for 15 h (HL‐60 and U937 cells) with 0.2 µg·mL^−1^ of a monoclonal anti‐Fas antibody (Sigma‐Aldrich, Stockholm, Sweden) or 40 µm H_2_O_2_ to induce apoptosis. To study effects of protease inhibitors, 1 µm of recombinant cystatins A, C, D, E/M, or chagasin was added at the same time as apoptosis was induced. Following incubation, cells were lysed with 100 µL lysis buffer and 25 μL of the lysate was added to a black, flat‐bottomed 96‐well microplate (Nunc A/S). After addition of 70 μL caspase buffer (10% sucrose, 0.1% CHAPS, 10 mm DTT, 100 mm Tris, pH 7.4) and the fluorogenic substrate Z‐DEVD‐NHMec (Sigma‐Aldrich) to a final concentration of 20 μm, the fluorescence was measured every minute for 120 min (Fluoroscan Ascent plate reader; LabSystems, Stockholm, Sweden) at excitation/emission wavelengths of 355/460 nm. The temperature was set to 37 °C, and all measurements were performed in triplicate. The total protein content in the lysates was measured by Coomassie Protein Assay. The fluorescence increase with time during the initial phase of the enzyme reaction was divided by the total protein content (mg) in the cell lysate to give the caspase‐3‐like activity expressed as FU·min^−1^·mg^−1^ of protein.

### Flow cytometry

U937 cells were seeded in a 12‐well plate at a density of 100 000 cells·mL^−1^ with addition of 0, 1, or 5 µm cystatin C or D for 24 h. The experiment was carried out with cells induced for apoptosis by hydrogen peroxide or with nonapoptotic cells. Apoptosis was assessed by staining the cells in 1× binding buffer (BD Biosciences, San Jose, CA, USA) with APC conjugated annexin V (BD Biosciences) and DAPI (0.5 μg·mL^−1^; BioLegend, San Diego, CA, USA), and analyzed using a FACS LSRFortessa (BD Biosciences).

### MTT viability assay

For dose–response experiments, U937 cells were seeded into a round‐bottomed 96‐well cell culture plate at a density of 10 000 cells·mL^−1^ and incubated with 0, 1, 3, or 9 µm of cystatin C or D for 24, 48, or 72 h. For other viability experiments, HL‐60, Jurkat, or U937 cells were seeded at the same density and incubated with 0 or 3 µm cystatin C or D for 48 h. Experiments were either conducted with nonapoptotic cells or with cells incubated with 40 µm hydrogen peroxide to induce apoptosis. MTT dye (5 mg·mL^−1^ ;Sigma‐Aldrich) was added to the cells after the initial incubation step before a new incubation in 37 °C for 4 h. The yellow MTT reagent is converted to purple water insoluble crystals of formazan by living cells only. The crystals were dissolved with 100% DMSO (Sigma‐Aldrich), and the absorbance was measured in a microplate reader at 540 nm [44, 45].

### Quantification of cystatins

U937 cells were seeded in 24‐well cell culture plates (500 000 cells/well) and incubated for 5 h in standard medium with either wild‐type cystatin C, W106F‐cystatin C, or (R24A,R25A)‐cystatin C at 1 µm final concentration. After incubation, cells were washed twice with PBS (Life Technologies Ltd.) and incubated for 30 min with 250 µL lysis buffer. Cell debris was removed by a centrifugation step before analysis.

ELISA was performed for the determination of cystatin C and D content in cell lysates. Cystatin C was analyzed in accordance with a previously described procedure [46]. The IgG fraction of a polyclonal rabbit anti‐(human cystatin C) antibody (antiserum 8206) was used to capture the antigen, followed by a secondary biotinylated monoclonal mouse anti‐(human cystatin C) antibody. A streptavidin‐HRP conjugate (Amersham Biosciences, Uppsala, Sweden) was utilized for detection of biotinylated antibody in the double‐sandwich ELISA, and finally the peroxidase substrate ABTS (Thermo Fisher Scientific) was added. For appropriate calibration curves, recombinant human cystatin C [42] was used.

Cystatin D was analyzed by a similar ELISA method. Wells were coated with 100 µL of 0.5 µg·mL^−1^ monoclonal mouse anti‐(human cystatin D) antibody (R&D Systems Inc., Minneapolis, MN, USA). For construction of a standard curve, highly purified recombinant human Arg26‐cystatin D [17] was used in dilutions ranging from 0.1 to 100 ng·mL^−1^. For the detection step, we used a biotinylated fraction of purified IgG from a polyclonal rabbit anti‐(human cystatin D) antiserum [17], followed by streptavidin‐HRP conjugate diluted 1 : 1000 (Amersham). OPD (Thermo Fisher Scientific) was used as a substrate, and absorbance was measured in a SpectraMax 340 PC Plate Reader (Molecular Devices, San Jose, CA, USA) at 490 nm.

The concentration of the cystatins was related to total protein concentration in the homogenates measured by Coomassie Protein Assay.

### Confocal laser scanning microscopy

U937 cells were seeded into a 6‐well culture plate (250 000 cells/well) and incubated for 5 h with 3 µm Alexa Fluor 568‐cystatin C or/and Alexa Fluor 488‐cystatin D, labeled, and purified according to recommendations by the fluorophore supplier (Molecular Probes). Then, the cells were fixed with 4% (v/v) paraformaldehyde for 10 min before nuclei were stained with 0.5 μg·mL^−1^ DAPI (Thermo Fisher Scientific). Cells were applied to coned microscope wells (35 mm ibiTreat µ‐Dish; Ibidi, Martinsried, Germany) and allowed to settle for 1 h. Confocal laser scanning microscopy (CLSM) of the cells incubated with fluorescently labeled cystatins was performed with a Zeiss LSM 800 microscope (Zeiss, Stockholm, Sweden) at ImaGene‐iT, Lund, Sweden. Settings of detection for Alexa Fluor 568, Alexa Fluor 488 and DAPI were based on fluorescence detection levels of control cells incubated without added fluorophores. The settings were kept through the analyses of all samples. Scans were made through the cells to ensure detection of intra‐ or extracellular immunofluorescence. Image documentation was made from at least three representative regions of each well.

For immunostaining, 10^6^ HL‐60, Jurkat, and U937 cells were seeded into wells of a 6‐well plate, with and without 5 µm cystatin D in 1 mL standard medium and incubated for 5 h. Then, the cells were fixed with 4% (v/v) paraformaldehyde for 10 min before permeabilization with 0.1% Triton X‐100 in PBS for 20 min and blocking with 1% (w/v) BSA in PBS containing 0.1% Triton X‐100 for 1 h. Immunostaining was performed with a primary polyclonal rabbit‐anti‐(human cystatin D) antiserum [17], diluted 1 : 200, and a secondary goat anti‐rabbit IgG Alexa Fluor 594‐conjugated antibody (Thermo Fisher Scientific), diluted 1 : 500. Cell nuclei were visualized by staining with DAPI (0.5 μg·mL^−1^). The samples were loaded into a µ‐Plate 96 well, transparent‐bottomed black plate (Ibidi) and then imaged with a Nikon A1R HD25 confocal microscope (Tokyo, Japan) equipped with Nikon A1 photomultiplier. Z‐stacks were composed in a total span of 10 µm with a Z‐step of 1 µm between each optical plane, in which magnifications of 40× and 100× were collected. nis‐elements Imaging Software, version 1.2 (Nikon) was used to visualize the cells. Maximum intensity projections of each picture were analyzed using imaris software version 9.5 (Bitplane, Belfast, UK).

## Conflict of interest

The authors declare no conflict of interest.

## Author contributions

SH performed the majority of experiments, participated in planning, interpretation of data and writing; HW planned, performed, and supervised experimental work, analyzed data and wrote; ME and MJ performed and interpreted flow cytometry experiments; MA planned and supervised experimental work, analyzed data, and coordinated writing of the paper.

## Data Availability

All data will be available from the corresponding author upon reasonable request. Enzyme used: caspase‐3 (EC 3.4.22.56).
